# Plastron Respiration Using Commercial Fabrics

**DOI:** 10.3390/ma7010484

**Published:** 2014-01-16

**Authors:** Shaun Atherton, Joseph C. Brennan, Robert H. Morris, Joshua D.E. Smith, Christopher A.E. Hamlett, Glen McHale, Neil J. Shirtcliffe, Michael I. Newton

**Affiliations:** 1 School of Science and Technology, Nottingham Trent University, Clifton Lane, Nottingham NG11 8NS, UK; E-Mails: joseph.brennan@ntu.ac.uk (J.C.B.); rob.morris@ntu.ac.uk (R.H.M.); jd.smith1791@gmail.com (J.D.E.S.); christopher.hamlett@ntu.ac.uk (C.A.E.H.); michael.newton@ntu.ac.uk (M.I.N.); 2 Faculty of Engineering and Environment, Northumbria University, Newcastle upon Tyne NE1 8ST, UK; E-Mail: glen.mchale@northumbria.ac.uk; 3 Hochschule Rhein-Waal, Marie-Curie-Str. 1, Kleve D-47533, Germany; E-Mail: neil.shirtcliffe@hochschule-rhein-waal.de

**Keywords:** plastron, hydrophobic, textile, respiration, underwater breathing

## Abstract

A variety of insect and arachnid species are able to remain submerged in water indefinitely using plastron respiration. A plastron is a surface-retained film of air produced by surface morphology that acts as an oxygen-carbon dioxide exchange surface. Many highly water repellent and hydrophobic surfaces when placed in water exhibit a silvery sheen which is characteristic of a plastron. In this article, the hydrophobicity of a range of commercially available water repellent fabrics and polymer membranes is investigated, and how the surface of the materials mimics this mechanism of underwater respiration is demonstrated allowing direct extraction of oxygen from oxygenated water. The coverage of the surface with the plastron air layer was measured using confocal microscopy. A zinc/oxygen cell is used to consume oxygen within containers constructed from the different membranes, and the oxygen consumed by the cell is compared to the change in oxygen concentration as measured by an oxygen probe. By comparing the membranes to an air-tight reference sample, it was found that the membranes facilitated oxygen transfer from the water into the container, with the most successful membrane showing a 1.90:1 ratio between the cell oxygen consumption and the change in concentration within the container.

## 1. Introduction

**Figure 1.**
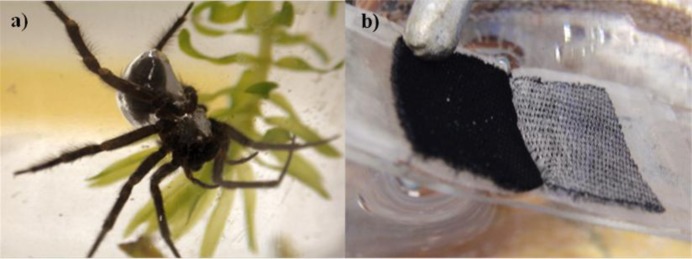
(**a**) Image of submerged diving bell spider reproduced from [8]; (**b**) submerged hydrophobic fabric, both showing the silvery sheen of a plastron.

Certain hydrophobic surfaces will retain an air layer, known as a plastron, on their surface when submerged underwater which signifies the Cassie-Baxter state of wetting [1]. The plastron is easily recognizable due to a silver sheen resulting from the reflections at the air-water interface. A variety of insect and arachnid species have been observed to support a plastron around part of their body while underwater. The plastron serves two purposes; firstly it acts as an oxygen reservoir allowing the creature to breathe whilst submerged and secondly it forms a physical gill enabling the creature to draw dissolved oxygen from the water around it. There is a water-vapour interface between the air layer and the surrounding water which allows gaseous diffusion to take place, by oxygen moving from the water to the air layer and carbon dioxide escaping out into the water [2,3]. This phenomenon was first observed by Chomstock in 1887 [4], where he found that the Corisa genera of the water-boatman was able to remain underwater far longer than would be expected, and was described in more detail by Crisp *et al*. [5]. Plastron respiration has also been observed in non-tracheate arthropods [6]. The Diving Bell Spider (Argyroneta aquatic), Figure 1, is unique amongst spiders as it is thought to live its entire life underwater [7,8]. The spider uses hydrophobic hairs to create a plastron around its abdomen and legs; it is then able to use this air layer to survive whilst moving around underwater. The name Diving Bell Spider comes from the way the spider will use its web to build an air pocket amongst submerged plants by bringing air down from the surface. This air pocket can vary in size from large enough to contain the entire spider to so small only the abdomen will fit inside it.

In the field of biomimetics the use of hydrophobic surfaces are common [9] and used for a variety of applications such as fog harvesting and transportation [10] and buoyant cargo carriers [11]. Much work has gone into studying plastron respiration [2,12–14] with some attempts at recreating it, such as the work done by Shirtcliffe *et al*. [15]. They produced a sol gel foam using methyltriethoxysilane, creating a hydrophobic structure with micron sized pores which when submerged underwater retained a plastron on its surface. A zinc/oxygen cell was used as a method of consuming oxygen and sealed within a cavity made from the sol gel. They found that the hydrophobicity of the material, coupled with a small pore size, kept the water out, while allowing for the exchange of oxygen between the cavity and the water. Many fabrics have been developed that are highly water-resistant with some exhibiting hydrophobic and superhydrophobic properties [16–18]. In this article a selection of fabrics, as well as porous polytetrafluoroethylene (PTFE), are investigated to determine their hydrophobicity, whether they are plastron bearing and the efficiency with which they transport oxygen across their surface.

## 2. Experimental Method

### 2.1. Materials

In order to mimic plastron respiration a range of porous, hydrophobic materials were used to mimic the effects of a physical gill. These included two types of porous PTFE, a PTFE thread seal tape (No Nonsense Ltd., Yeovil, UK) and expanded PTFE as used in Gore-Tex (Gore and Associates Ltd., Livingstone, Scotland). There were also a number of hydrophobic woven fabrics, including a hydrophobized nylon (Toray Textiles, Mansfield, UK), a hydrophobized polyester (Toray Textiles, Mansfield, UK) and a hydrophobized deep pile polyester fabric (Furtech, Glynneath, Wales, UK), all of which were treated with Granger’s Extreme Wash-in (Granger’s, UK), plus another polyester which had been hydrophobized (Furtech, Glynneath, Wales, UK). Several samples were also provided by Speedo (Speedo International, Nottingham, UK) from their swimwear range, including Speedo Aquablade^®^, Speedo LZR Pulse^®^, Speedo Fastskin^®^ and Speedo Endurance^®^.

### 2.2. Characterisation of Materials

To categorize the materials the static, advancing and receding contact angles were measured. Using a Kruss Drop Shape Analysis System (Kruss, Hamburg, Germany) 5 μL of water was dispensed onto the surface of each material, the static contact angle was then measured. The droplet was increased in volume up to 20 μL and then decreased back to 5 μL. As the droplet changes in size, the contact point will initially stay static before eventually moving to a new contact point. The contact angle was measured immediately prior to the contact point moving as the droplet increased or decreased in volume, giving the advancing and receding contact angles respectively. Due to the rough nature of some of the surfaces, a precise contact angle can be hard to achieve. This is due to the difficulty in finding the exact contact point of the water on the surface.

Samples of the materials were imaged using a scanning electron microscope (SEM). To prepare them for imaging a 75 nm layer of gold was applied using an Emitech K575X sputter coater (Qourum Technologies Ltd. East, Grinstead, UK). The samples were then imaged using a Jeol JSM-840A SEM (Jeol Ltd., Tokyo, Japan). Where possible image processing was used to measure the pore size of the material, this was done using ImageJ [19].

Images were taken of the plastron layer using a confocal microscope (Leica DM-IRBE inverted confocal microscope). For this the samples were submerged in water and the focal plane of the microscope was moved from the surface of the sample to above the top of the plastron layer. This allowed the coverage of the plastron to be measured.

### 2.3. Preparation of Samples

**Figure 2.**
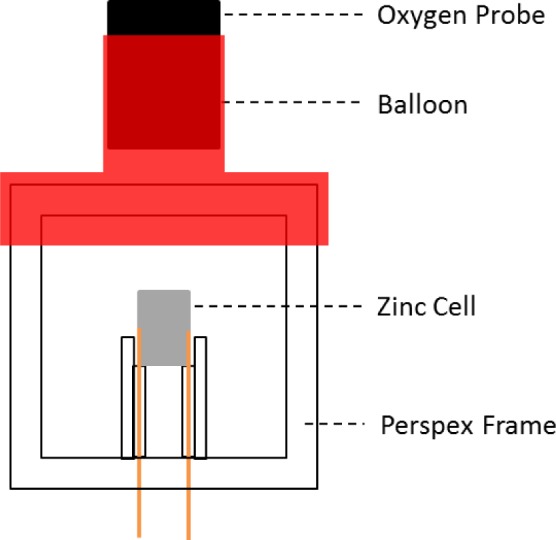
Diagram of respiration box.

In order to test the effectiveness of the membranes for plastron respiration a box was constructed from each material with a volume of 16.57 cm^3^ and a surface area of 23 cm^2^, Figure 2. First a frame was made from Perspex sheets using a laser cutter (40W M-300, Universal Laser Systems, Scottsdale, AZ, USA) which the membrane could be attached to. The material was attached to four sides of the frame using Araldite^®^ two part epoxy (Bostik, Leicester, UK). The bottom of the box was solid Perspex with two holes cut out to pass wires through, which was sealed around using Araldite. The top was sealed over with a balloon, also attached using Araldite^®^, to provide a water tight seal for an oxygen probe. A zinc/oxygen cell (DA13 Zinc-air Coin Cell, Duracell, UK) was held within the box. The cell generates electricity by using oxygen from the air to oxidize the zinc, and so would consume the oxygen within the box. Wires coming from the box connected the cell into a constant current circuit (CCC), consisting of an LM334 adjustable current source (RS Components, Corby, UK) in parallel with a 10 Ω resistor and a 200 Ω resistor in series with both. A voltmeter was used to measure the voltage across the cell and an ammeter to measure the current through the CCC. In order to measure the oxygen concentration inside the box an Extech 407510 oxygen meter (Extech Instruments, Nashua, NH, USA) was used. The sensing end of the probe was sealed inside the box using the balloon to make a water tight seal. Data from the three measurement devices was logged to a computer, using proprietary software from Extech for the probe and LabView (National Instruments, Austin, TX, USA) for the voltmeter and ammeter.

### 2.4. Measurement of Oxygen Levels

To investigate the effectiveness of each material the box, with cell connected into CCC and oxygen probe sealed inside, was submerged 10 cm underwater. A silvery sheen would indicate the presence of a plastron. The current and voltage of the cell and the oxygen concentration within the box were measured over the course of 6 h. During this time a Tetratec APS100 air pump (Tetra, Melle, Germany) was used to keep the water equilibrated with the atmosphere and to provide some stirring. The oxygen consumption rate could be directly calculated from the current supplied by the cell, finding the total consumed by the cell over the 6 hour experiment. This was then compared with the change in oxygen partial pressure in the container, as measured by the oxygen probe. Therefore, by monitoring the oxygen levels within the box, it can be determined whether or not the oxygen levels within the box get replenished from plastron respiration across the membrane. As well as running this experiment underwater the experiment was also performed in air to see if the oxygen replenishment rate is affected by the fabric alone.

## 3. Results and Discussion

### 3.1. Characterisation of Materials

#### 3.1.1. SEM

**Figure 3.**
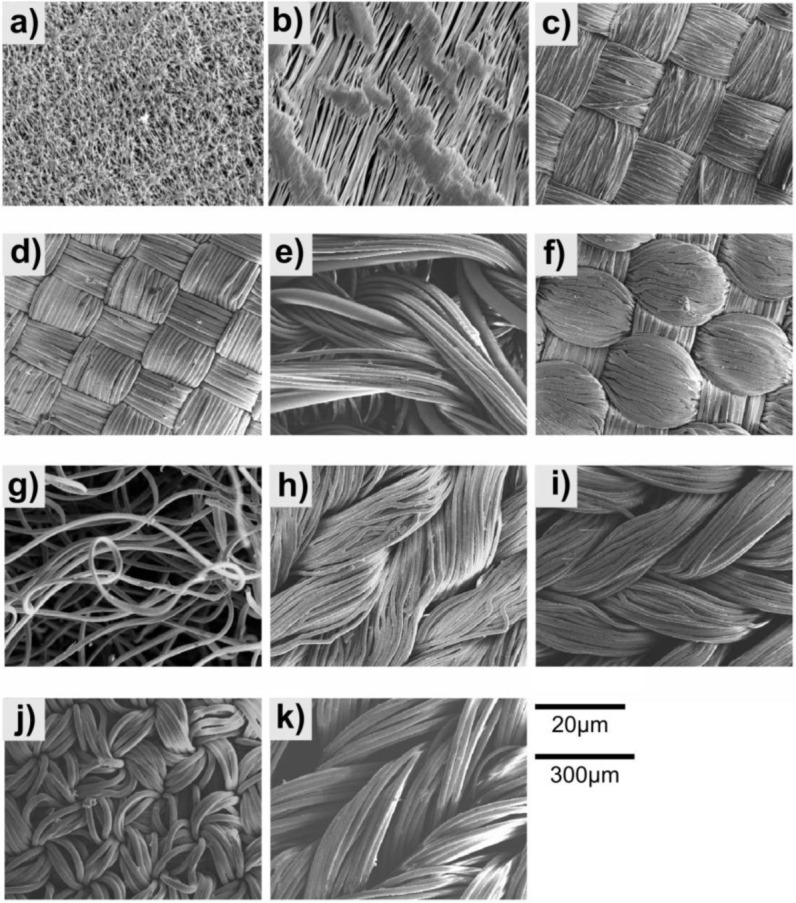
SEM images at 2000× magnification of (**a**) Gore-Tex; and (**b**) PTFE Tape. SEM images at 150× magnification of (**c**) Furtech Polyester; (**d**) Toray Nylon; (**e**) Deep Pile Polyester Reverse; (**f**) Toray Polyester; (**g**) Deep Pile Polyester; (**h**) Speedo Fastskin^®^; (**i**) Speedo Aquablade^®^; (**j**) Speedo LZR Pulse^®^; and (**k**) Speedo Endurance^®^. The 20 μm scale bar is for images (a) and (b). The 300 μm scale bar is for images (**c**–k).

Figure 3 shows SEM images of the tested materials. The materials used consisted of either woven fabrics or porous polymer sheets. The PTFE tape and Gore-Tex are both single sheets with pores through which gas can pass. The PTFE tape has a maximum pore size of 14.8 μm^2^ and the pores making up 10.1% of the surface area. The Gore-Tex has a maximum pore size of 12.0 μm^2^ and the pores make up 6.2% of the surface area. The other materials are made up of several fibres woven together. The Furtech Polyester appeared to have a loose weave with a fibre diameter of 13.6 μm. The Toray Nylon had the most regular and tight weave of the fabrics and a fibre diameter of 14.5 μm. The Deep Pile Polyester Reverse had a fibre diameter of 13.9 μm, which would also be the case for the Deep Pile Polyester. The pile on the Deep Pile Polyester was made up of 18.1 μm fibres. The Toray Polyester is less tight weave than the Toray Nylon but has a similar fibre diameter 14.0 μm. All of the Speedo fabrics appear to be a more irregular weave, with the Fastskin and Aquablade having fibre diameters of 5.92 μm and 5.75 μm respectively and the LZR Pulse and Endurance having diameters of 10.30 μm and 10.99 μm respectively.

## 3.1.2. Contact Angles, Thickness and Density

Table 1 shows the static, advancing and receding contact angles for the materials tested. A material is considered hydrophobic if it has a static contact angle ≥90° and super-hydrophobic if the contact angle is ≥150°. None of the materials had contact angles high enough to be considered superhydrophobic, with the highest being just short at 144° for the Deep Pile Polyester and the Furtech Polyester, but all were well within the range to be considered hydrophobic, the lowest value being for the Gore-Tex fabric at 117°. The difference between the advancing and receding contact angles shows the range of angles a droplet on the surface can take and gives a measure of the mobility of water across the surface of the material [16]. Contact angle hysteresis is the difference between the advancing and receding contact angles. The materials showed a wide range of hysteresis values, the lowest of which being the Deep Pile Polyester at 20°, suggesting a material with high water mobility across the surface, and the highest being the Furtech Polyester at 144°, suggesting a material with low water mobility across the surface. The high hysteresis values are due to the water being pinned to the surface. This means that the contact point did not recede during the retraction of water.

Table 1 also shows the thickness and densities of the fabrics. The thickness of the materials was measured using the same side on microscope as was used to take the contact angle images (Kruss, Hamburg, Germany). The mass of the sample was measured by cutting out a 100 cm^2^ piece of each sample and weighing it using a set of laboratory scales. The thinnest sample was the Toray Nylon with a thickness of 0.07 mm. This was closely followed by the PTFE tape and Toray Polyester at 0.10 mm and 0.11 mm respectively. The thickest sample was the Deep Pile Polyester at 2.18 mm thick. The PTFE tape has the highest density at 1200 kg/m^3^ and the least dense sample was the Deep Pile Polyester with a density of 90 kg/m^3^.

## 3.1.3. Plastron Properties and Final Oxygen Level

All of the materials exhibited a visible plastron when submerged underwater. This was confirmed with confocal microscopy that showed plastrons of various thickness and varying coverage for the samples. As many of the materials were challenging to image, Table 2 includes a number that is representative of the coverage of the plastron as seen by the confocal microscope with full plastron coverage being the best at 5 and no visible plastron at 1. No plastron was visible on the confocal microscope images of PTFE tape which is most likely due to the low reflectivity of any thin plastron layer and the high reflectivity of the white tape. However, it is possible to visually observe a large reflective layer across the whole surface of the PTFE tape and for this reason we have given PTFE tape a numerical value of 5. An ideal fabric would be one with a low density, low thickness and large plastron coverage. It is expected that the plastron breathing properties of a material should be proportional to the plastron coverage of the surface and be inversely proportional to the thickness and the density of the material. This result is shown in Table 2 for each material tested. The material with the highest Plastron coverage/(Density × Thickness) was the Speedo LZR Pulse^®^ fabric. This came out with a value of 45 ± 10 and was also the material with the highest %O_2_ after the plastron breathing experiments. The material with the lowest Plastron coverage/(Density × Thickness) value was the Speedo Aquablade^®^ fabric. This had a value of 4 ± 1 and had the second lowest %O_2_ after the plastron breathing experiments.

### 3.2. Measurement of Oxygen Levels

**Figure 4.**
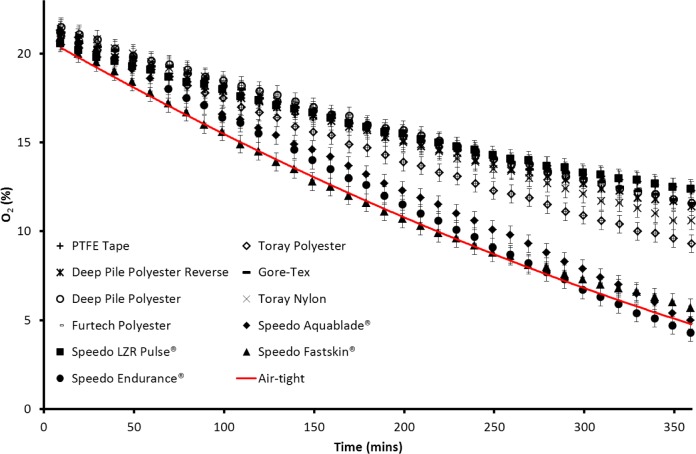
The change in oxygen concentration with time measured with the oxygen meter for the materials tested and for an air-tight container (solid line).

The fabric boxes were ran in air to see how well the oxygen could be transported through the fabrics. The lowest value of %O_2_ after 6 h was 19.7% for the Furtech Polyester sample. The highest value was 20.5% for the Speedo LZR Pulse^®^ box. This shows that all of the boxes had similar oxygen mobility when tested in air and that this is not a limiting factor in the plastron breathing experiment. Figure 4 shows the oxygen concentration changes with time both within the boxes formed by the various membranes and an air-tight container acting as a reference sample (solid line). For most of the materials tested there is a clear difference in the rate at which the oxygen level decreases compared to the air-tight sample. The Speedo Aquablade^®^, Speedo Fastskin^®^ and Speedo Endurance^®^ materials are the closest to the air-tight result and therefore represent the lowest oxygen transference from the surrounding water. The other materials all show a considerably slower rate of oxygen decrease than the air-tight container, with the Speedo LZR Pulse^®^ showing the slowest oxygen decrease, representing a greater amount of oxygen transference. The oxygen partial pressure/percentage in the air-tight container had reached 4.7% by the end of the experiment. The zinc cell in the air-tight container consumes the fixed amount of oxygen sealed within and would be expected to continue until the zinc-oxygen reaction is no longer able to power the circuit, this would be expected to reach zero given a longer experiment. Oxygen partial pressures/percentages in plastron bearing fabric containers largely reached values higher than 4.7%. The Speedo Endurance^®^ reached a final value of 4.3%, taking into account experimental errors this is comparable to the air-tight container. The Speedo Aquablade^®^ and Fastskin^®^ had final values close to the air-tight box at 5% and 5.7% respectively. The Speedo LZR Pulse^®^ and PTFE Tape had the highest final values at 12.4% and 12.3% respectively. The difference in the O_2_ decrease shows that the containers are able to draw oxygen from the dissolved oxygen within the water, and are therefore working as an external gill.

**Figure 5.**
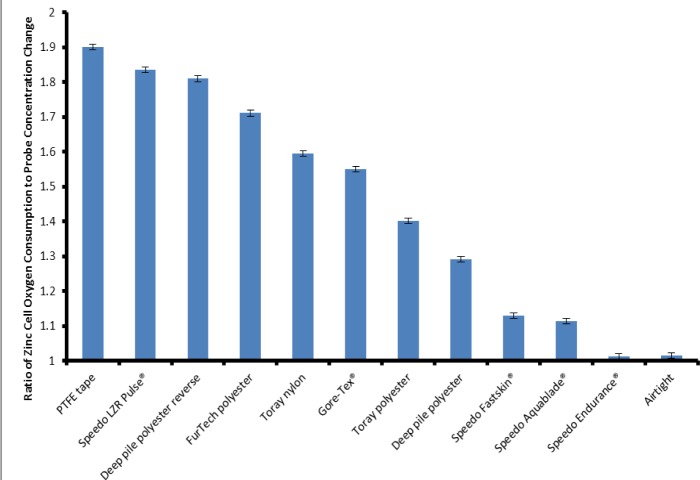
The ratio of oxygen consumed by zinc cell to the decrease in oxygen concentration as measured by the oxygen probe.

The zinc cell was calculated to consume oxygen at a rate of approximately 500 μL·h^−1^. Figure 5 shows the ratio of the oxygen consumed by the zinc cell to the oxygen decrease as measured by the probe for each individual sample. This allows us to compare the various materials and their ability to support plastron respiration. The Air-tight container shows a ratio of 1.02:1, this is close to 1:1 which is to be expected as there is no replenishing of the oxygen from the water. In agreement with the data shown in Figure 4, the Speedo Endurance proved the worst material for oxygen transfer with a ratio comparable to the Air-tight box. The PTFE tape proved to be the best material for plastron respiration, showing a ratio of 1.90:1 when comparing the O_2_ used up by the zinc cell and O_2_ decrease measured by the probe. This shows that over the course of the experiment the O_2_ within the container was being replenished by the dissolved O_2_ in the water. Of the woven fabrics the Speedo LZR Pulse^®^ performed the best. Only the Speedo Endurance^®^ appeared to allow no oxygen to transfer across the membrane, showing a ratio comparable to the Air-tight container. The other materials showed varying degrees of oxygen exchange, all of them showing some evidence of oxygen transfer with ratios falling between the air-tight container and the PTFE tape.

**Figure 6.**
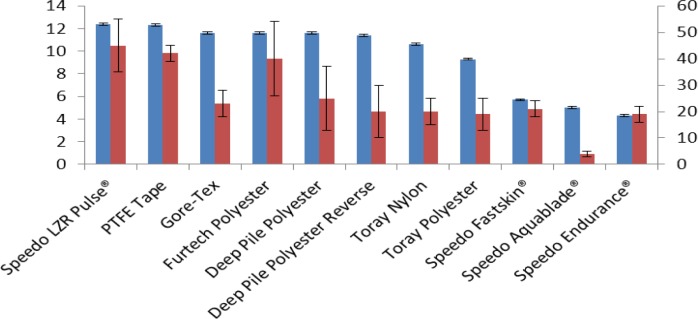
The %O_2_ in the fabric boxes after 6 h (blue bar and left *y*-axis) and Plastron coverage/(Density *×* Thickness) (red bar and right *y*-axis) of the fabrics.

Figure 6 shows the %O_2_ in the sample boxes after 6 h and the Plastron coverage/(Density × Thickness) value for each sample arranged with the highest %O_2_ first then in descending order. The graph confirms that a higher Plastron coverage/(Density × Thickness) value is an indicator to a good sample for plastron respiration with Speedo LZR Pulse^®^ coming out with the highest value and the highest %O_2_ after 6 h and the lowest value for the Speedo Aquablade^®^ fabric which had the second lowest %O_2_ after 6 h.

## 4. Conclusions

We have shown that hydrophobic fabrics and porous polymers can be used to mimic underwater breathing. Of the materials tested the best oxygen transfer rates were shown by the PTFE tape and the Speedo LZR Pulse*^®^*. We have shown that by knowing the thickness, density and plastron coverage of a sample, an estimate of its performance in the plastron breathing experiment can be achieved.

The effectiveness of the material for oxygen transfer is related to the physical dimensions of the material itself. This is supported by looking at the two different types of PTFE, the tape and the Gore-Tex. Both membranes showed very different levels of oxygen transfer. The lower transfer rate of the Gore-Tex may be due to the smaller pore size and the lower percentage of the surface made up of pores. The nature of the fabric samples made measuring the pore size and porosity of the materials unsuccessful and no clear link was found between fibre size and oxygen transfer.

In the work of Shirtcliffe *et al*. [15], after a period of 6 h their sol gel container had reached an oxygen concentration of ≈14% and eventually reaches an equilibrium value of ≈12%. The materials in this study reach a lower concentration after the same 6 hour period. However, the oxygen consumption rate in their experiment was 246 μL·h^−1^, much lower than the ≈500 μL·h^−1^ consumption caused by the cell in this study.

## Figures and Tables

**Table 1.** Measurements of the static, advancing and receding contact angles and the contact angle hysteresis for the materials used in the study. P in the receding contact angle column denotes that the water was pinned to the surface of the material. The thickness and densities of each fabric are also shown.

Material	Thickness (mm)	Density (kg/m^3^)	Static Contact angle (°)	Advancing Contact Angle (°)	Receding Contact Angle (°)	Plastron visible on surface
Gore-Tex	0.29 ± 0.03	380 ± 20	115 ± 2	117 ± 2	P	Yes
Furtech Polyester	0.100 ± 0.003	1200	126	144	P	Yes
Toray Nylon	1.21 ± 0.05	180	103	129	P	Yes
Toray Polyester	0.36 ± 0.05	210	83	106	P	Yes
Deep Pile Polyester	2.18 ± 21	90	111	127	107 ± 2	Yes
Deep Pile Polyester Reverse	2.18 ± 21	90	116	144	77	Yes
PTFE Tape	0.07 ± 0.01	720	127	139	79	Yes
Speedo Aquablade®	0.11 ± 0.02	940	112	123	P	Yes
Speedo LZR Pulse®	0.67 ± 0.04	360	115	120	88	Yes
Speedo Fastskin®	0.62 ± 0.04	360	122	133	101	Yes
Speedo Endurance®	0.55 ± 0.03	390	110	118	P	Yes

**Table 2.** Values for the plastron coverage of the samples, the plastron coverage/(density × thickness) values and the %O_2_ levels in the box after 6 h submerged.

Material	Proportion of plastron covering the surface as imaged by confocal microscope	Plastron coverage numerical value	Plastron coverage/(Density *×* Thickness)	%O_2_ for box in water after 6 h
Speedo LZR Pulse^®^	Covering surface	5	45 ± 10	12.4 ± 0.1
PTFE Tape	None visible	5	42 ± 3	12.3
Gore-Tex	Covering surface	5	23 ± 5	11.6
Furtech Polyester	Plastron between fabric weave	3	40 ± 14	11.6
D P Polyester	Covering surface	5	25 ± 12	11.6
D P Polyester Reverse	Partial surface coverage	4	20 ± 10	11.4
Toray Nylon	None visible	1	20 ± 5	10.6
Toray Polyester	Bubbles on surface	2	19 ± 6	9.3
Speedo Fastskin^®^	Covering surface	5	21 ± 3	5.7
Speedo Aquablade^®^	None visible	1	4 ± 1	5.0
Speedo Endurance^®^	Partial surface coverage	4	19 ± 3	4.3
